# Extracorporeal versus conventional cardiopulmonary resuscitation for refractory out-of-hospital cardiac arrest: a secondary analysis of the Prague OHCA trial

**DOI:** 10.1186/s13054-022-04199-3

**Published:** 2022-10-27

**Authors:** Daniel Rob, Jana Smalcova, Ondrej Smid, Ales Kral, Tomas Kovarnik, David Zemanek, Petra Kavalkova, Michal Huptych, Arnost Komarek, Ondrej Franek, Stepan Havranek, Ales Linhart, Jan Belohlavek

**Affiliations:** 1 2nd Department of Medicine – Department of Cardiovascular Medicine, First Faculty of Medicine, Charles University and General University Hospital in Prague, U Nemocnice 2, 128 00 Prague, Czech Republic; 2 Czech Institute of Informatics, Robotics and Cybernetics (CIIRC), Czech Technical University in Prague, Prague, Czech Republic; 3 Department of Probability and Mathematical Statistics, Faculty of Mathematics and Physics, Charles University in Prague, Prague, Czech Republic; 4 Emergency Medical Service Prague, Prague, Czech Republic

**Keywords:** Out-of-hospital cardiac arrest, Extracorporeal life support, Extracorporeal membrane oxygenation, Extracorporeal cardiopulmonary resuscitation, Return of spontaneous circulation

## Abstract

**Background:**

Survival rates in refractory out-of-hospital cardiac arrest (OHCA) remain low with conventional advanced cardiac life support (ACLS). Extracorporeal life support (ECLS) implantation during ongoing resuscitation, a method called extracorporeal cardiopulmonary resuscitation (ECPR), may increase survival. This study examined whether ECPR is associated with improved outcomes.

**Methods:**

Prague OHCA trial enrolled adults with a witnessed refractory OHCA of presumed cardiac origin. In this secondary analysis, the effect of ECPR on 180-day survival using Kaplan–Meier estimates and Cox proportional hazard model was examined.

**Results:**

Among 256 patients (median age 58 years, 83% male) with median duration of resuscitation 52.5 min (36.5–68), 83 (32%) patients achieved prehospital ROSC during ongoing conventional ACLS prehospitally, 81 (32%) patients did not achieve prehospital ROSC with prolonged conventional ACLS, and 92 (36%) patients did not achieve prehospital ROSC and received ECPR. The overall 180-day survival was 51/83 (61.5%) in patients with prehospital ROSC, 1/81 (1.2%) in patients without prehospital ROSC treated with conventional ACLS and 22/92 (23.9%) in patients without prehospital ROSC treated with ECPR (log-rank *p* < 0.001). After adjustment for covariates (age, sex, initial rhythm, prehospital ROSC status, time of emergency medical service arrival, resuscitation time, place of cardiac arrest, percutaneous coronary intervention status), ECPR was associated with a lower risk of 180-day death (HR 0.21, 95% CI 0.14–0.31; *P* < 0.001).

**Conclusions:**

In this secondary analysis of the randomized refractory OHCA trial, ECPR was associated with improved 180-day survival in patients without prehospital ROSC.

*Trial registration*: ClinicalTrials.gov Identifier: NCT01511666, Registered 19 January 2012.

## Background

Out-of-hospital cardiac arrest (OHCA) is one of the leading causes of death in Western countries [1]. Patients without prehospital return of spontaneous circulation (ROSC) bear a grave prognosis with survival rates as low as 4% [2–4]. An increasing number of cardiac arrest centers worldwide have established a collaboration with emergency medical services using early transport from the field and extracorporeal life support (ECLS) implantation during ongoing cardiopulmonary resuscitation (CPR) when ROSC is not achieved conventionally, a method called extracorporeal cardiopulmonary resuscitation (ECPR). However, the current 2020 American Heart Association as well as the recent European Resuscitation Council guidelines provide a weak recommendation for ECPR which may be considered as a rescue method in selected patients when conventional CPR is failing, with low certainty of evidence [5, 6]. In addition, both guidelines highlighted the need for further research to define patients who would benefit from this intervention most [5, 6].

To date, two prospective randomized trials on ECPR in refractory OHCA were published, both testing “load and go” strategy with in-hospital ECLS cannulation. The first is the ARREST trial [7] which randomized 30 patients with refractory ventricular fibrillation only and was prematurely stopped due to superiority of ECPR and showed that ECPR is a feasible rescue option after prolonged unsuccessful ACLS, where standard approach has negligible chance for success [7]. The second prospective trial is the recently published Prague OHCA study [8] which enrolled 256 patients during on-scene ongoing ACLS to invasive arm (including intra-arrest transport for in-hospital ECPR and immediate invasive assessment) or standard ACLS. The invasive treatment did not significantly improve survival with good neurologically outcome at 180 days compared to standard ACLS in the intention to treat analysis but showed a beneficial effect of the invasive approach in 30-day neurological outcome and a subgroup of patients with prolonged CPR over 45 min [8]. Importantly, the anticipated statistical scenario of expected benefit provided by invasive approach was not reached due to higher-than-expected survival in the standard group [8]. Further, crossovers were allowed and 4 out of 10 (40%) patients crossed from standard to invasive ECPR treatment survived 180 days [8]. In addition, part of the prehospitally randomized patients in both arms experienced ROSC before reaching the hospital and thus were not candidates for ECPR [8]. All these factors might have influenced the effect of the ECPR treatment in the intention to treat analysis. Therefore, we performed this secondary analysis of the Prague OHCA study to evaluate whether successful ECPR might have been associated with improved outcomes.

## Methods

### Population and study design

This study is a secondary analysis of the Prague OHCA study, a randomized clinical trial which was conducted at a single center in Prague, Czech Republic, from March 1, 2013, to October 25, 2020. Adult patients resuscitated for witnessed OHCA of presumed cardiac etiology after at least 5 min of ACLS were eligible for enrollment in the trial. A web-based secured randomization system was used to assign patient number and intervention group prehospitally during ongoing CPR in the field. The methodology and results of the intention to treat analysis were published in detail elsewhere [8, 9].

**Fig. 1:**
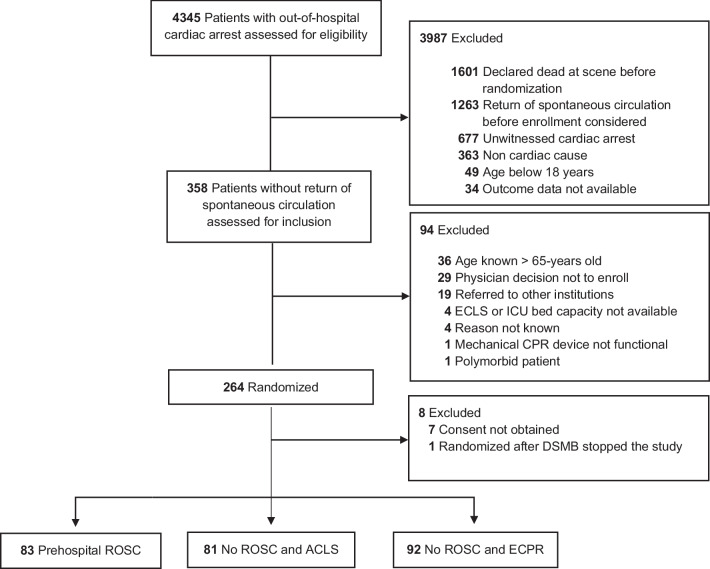
Modified consort flow diagram of the Prague OHCA study. *ACLS* advanced cardiac life support, *CPR* cardiopulmonary resuscitation, *DSMB* data safety monitoring board, *ICU* intensive care unit, *ECLS* extracorporeal life support, *ECPR* extracorporeal membrane resuscitation, *ROSC* return of spontaneous circulation

In the present analysis, all 256 enrolled patients were included and pooled into three groups (regardless of their original randomization assignment) according to their prehospital ROSC and ECPR status (Fig. 1). The first group (prehospital ROSC) is formed by all patients who achieved prehospital ROSC and thus were not candidates for ECPR. The second group is formed by all patients without prehospital ROSC despite prolonged ACLS who did not receive ECPR. This group includes patients who died during prolonged ACLS in the field as well as the group of patients admitted to the hospital who died during ACLS or achieved ROSC in the hospital. Finally, the third group is formed by all patients who received ECPR after arrival to the hospital.

The original study as well as secondary analyses was approved by the Institutional Review Board of the General University Hospital and First Faculty of Medicine, Charles University in Prague (192/11 S-IV). Each participant’s legal representative was informed of the participant’s study enrollment and was asked for written informed consent as soon as possible. All patients who regained normal neurological function were asked to provide their written consent regarding the use of their data. Consent requirements were waived for patients who died at the scene and never reached the hospital and for participants without known legal representatives. The research was carried out in accordance with requirements stated above (192/11 S-IV) and the Helsinki Declaration of 1964, revised in 2008.

### Outcomes

The primary outcome of the current analysis was all-cause 180-day survival. The secondary outcome was good neurological outcome at 180 days. A CPC of 1–2 was considered a good neurological outcome, and a CPC of 3–5 was considered a poor neurological outcome.

### Statistical analysis

The continuous data were tested to normal distribution by Shapiro–Wilk test. Categorical values are expressed as count and percentage, and the continuous variables are expressed as median and intra-quartile range. The Kruskal–Wallis test was used to compare the continuous values over all groups. Categorical values were tested using the chi-square test over all groups. Survival rates were compared using Kaplan–Meier analysis and Cox proportional hazards regression. Multivariate Cox proportional hazard model included all enrolled patients, and variables were age, sex, initial rhythm, prehospital ROSC status, time from collapse to EMS arrival, resuscitation time, place of cardiac arrest, percutaneous coronary intervention (PCI) status and ECPR status. A 2-sided *p* < 0.05 was considered statistically significant. Overall statistical analyses were performed with MedCalc® Statistical Software version 20.014 (MedCalc Software Ltd, Ostend, Belgium; https://www.medcalc.org; 2021), and the Cox proportional hazards model [10] analysis was performed using the R (R Core Team, 2021) software, version 4.1.0 (2021–05-18).

## Results

### Baseline characteristics

Baseline characteristics of patients according to the ROSC and ECPR status are presented in Table 1. There were no differences in age, sex, medical history or location of cardiac arrest between the three analyzed groups. Importantly, patients without prehospital ROSC with ACLS only compared to the ECPR group and prehospital ROSC group had significantly less common initial shockable rhythm (44.4% vs. 62% vs. 75.9%, *p* < 0.001) and consequently received more adrenalin doses (median 6 vs. 4 vs. 3, *p* < 0.001) and less defibrillations prehospitally (median 1 vs. 3 vs. 3, *p* = 0.02) (Table 1). Further, patients without ROSC treated with ACLS only as well as the ECPR group had much longer CPR times compared to patients with prehospital ROSC (median 66 and 60 vs. 31 min, *p* < 0.001) (Table 1).

**Table 1:** Baseline and resuscitation characteristics

Parameter	Prehospital ROSC (n = 83)	No ROSC and ACLS (n = 81)	No ROSC and ECPR (n = 92)	*P* value
Age (years)	55 (49–63.8)	58 (48–66)	58.5 (45–65)	0.69
Sex				
Woman	14 (16.9%)	14 (17.3%)	16 (17.4%)	1.0
Man	69 (83.1%)	67 (82.7%)	76 (82.6%)	
Medical history				
Hypertension	38 (48.1%)	13 (43.3%)	38 (46.3%)	0.9
Coronary artery disease	14 (17.9%)	5 (17.2%)	15 (18.8%)	0.98
Chronic heart failure	4 (5.1%)	3 (11.5%)	9 (11.1%)	0.35
Diabetes	14 (17.9%)	9 (31%)	13 (16.3%)	0.21
Chronic kidney disease	3 (3.8%)	0 (0%)	2 (2.5%)	0.58
COPD	7 (9.0%)	1 (3.8%)	2 (2.5%)	0.19
ICD implanted	0 (0.0%)	1 (2.7%)	2 (2.2%)	0.37
Location of cardiac arrest				
Home	26 (31.3%)	24 (29.6%)	26 (28.3%)	0.15
Public place	38 (45.8%)	29 (35.8%)	31 (33.7%)	
EMS	4 (4.8%)	12 (14.8%)	20 (21.7%)	
Health facility	0 (0%)	1 (1.2%)	1 (1.1%)	
Car	2 (3.6%)	5 (6.2%)	7 (7.6%)	
Hotel	4 (4.8%)	5 (6.2%)	1 (1.1%)	
Workplace	8 (9.6%)	5 (6.2%)	6 (6.5%)	
Initial rhythm				
VF	63 (75.9%)	36 (44.4%)	57 (62%)	** < 0.001**
Asystole	14 (16.9%)	23 (28.4%)	18 (19.6%)	
PEA	6 (7.2%)	22 (27.2%)	17 (18.5%)	
Time of CPR (time to death/ROSC or ECPR) (min)	31 (24–39.8)	66 (46–82.3)	60 (51–70)	**< 0.001**
Bystander CPR	81 (97.6%)	80 (98.8%)	91 (98.9%)	0.75
Time from collapse to EMS arrival (min)	9 (7–11)	9 (6–12)	8 (6–10)	0.6
Time from collapse to ACLS (physician arrival) (min)	11 (8.3–14)	11 (8–14.3)	10 (6–13)	0.06
Time from collapse to randomization (min)	24 (19–29.8)	26 (21–34.3)	24.5 (19.5–30)	0.27
Time to ECLS (min)	NA	NA	61 (55–70)	NA
Time of implantation (door to ECLS) (min)	NA	NA	12 (11–14)	NA
Number of epinephrine doses prehospitally (mg)	3 (2–4.8)	6 (5–9)	4 (2–6)	**< 0.001**
Dose of amiodarone prehospitally (mg) *	300 (0–300)	225 (0–300)	300 (0–300)	0.6
Number of defibrillations prehospitally	3 (2–5)	1 (0–4)	3 (0–6)	**0.02**

Highlighted in bold are the values which are statistically significant (less than 0.05)

*For patients with initial VF

*ACLS* advanced cardiac life support, *CPR* cardiopulmonary resuscitation, *COPD* chronic obstructive pulmonary disease, *ECPR* extracorporeal cardiopulmonary resuscitation, *ECLS* extracorporeal life support, *EMS* emergency medical service, *ICD* implantable cardioverter defibrillator, *PEA* pulseless electrical activity, *ROSC* return of spontaneous circulation, *VF* ventricular fibrillation

### Hospitalization characteristics, procedures, and cause of death

Admission characteristics and in-hospital interventions are described in Table 2. As most of the patients without prehospital ROSC treated with ACLS only died during initial CPR or the first hours after hospital admission (Table 2), they received less target therapeutic hypothermia (TTM) (28.6% vs. 97.8% vs. 94%, *p* < 0.001) and coronary angiography (CAG) (40% vs. 97.8% vs. 94%, *p* < 0.001) than patients treated with ECPR and patients with prehospital ROSC. Accordingly, patients without ROSC treated with ACLS only died mostly due to refractory cardiac arrest and patients treated with ECPR died primarily due to multiorgan dysfunction syndrome and brain death (Table 2). Only one patient in the prehospital ROSC group received ECLS for an arrhythmic storm with cardiogenic shock during hospitalization (Table 2). Further, patients treated with ECPR had a significantly higher rate of bleeding complications and a longer stay in the intensive care unit compared to others (Table 2).

**Table 2:** Hospitalization characteristics, procedures and cause of death

Parameter	Prehospital ROSC (n = 83)	No ROSC and ACLS (n = 81)	No ROSC and ECPR (n = 92)	*P* value
Admitted to the hospital	83 (100%)	35 (43.2%)	92 (100%)	**< 0.001**
Achieved ROSC	83 (100%)	9 (11.1%)	NA	**0.002**
Laboratory on admission				
pH	7.13 (7–7.19)	6.85 (6.75–6.91)	6.86 (6.75–6.98)	** < 0.001**
Lactate (mmol/L)	8.2 (6.2–11.5)	13.6 (11.1–17.5)	13.7 (10.95–17.0)	**< 0.001**
ECLS therapy	1 (1.2%)*	0	92 (100%)**	**< 0.001**
TTM used	78 (94%)	10 (28.6%)	90 (97.8%)	**< 0.001**
Coronary angiography	78 (94%)	14 (40%)	89 (97.8%)	** < 0.001**
PCI	37 (47.4%)	4 (28.6%)	51 (57.3%)	0.1
Successful	31 (83.8%)	2 (50%)	47 (92.2%)	**0.04**
Unsuccessful	6 (16.2%)	2 (50%)	4 (7.8%)	
Cause of death				
Refractory arrest	1 (2.9%)	72 (90%)	7 (9.9%)	**< 0.001**
Brain death	9 (26.5%)	2 (2.5%)	19 (26.8%)	
MODS	17 (50%)	4 (5%)	31 (43.7%)	
Cardiogenic shock	3 (8.8%)	1 (1.3%)	10 (14.1%)	
UNK	4 (11.8%)	0 (0%)	1 (1.4%)	
Bleeding	0 (0%)	1 (1.3%)	3 (4.2%)	
WLST	13 (15.7%)	2 (2.5%)	20 (21.7%)	** < 0.001**
Complications				
Bleeding—any***	5 (6.1%)	1 (8.3%)	40 (44%)	** < 0.001**
Fatal	0 (0%)	1 (100%)	3 (7.5%)	**0.03**
Intracranial	1 (20%)	0 (0%)	9 (22%)	
Overt	4 (80%)	0 (0%)	28 (70%)	
Organ lacerations	2 (2.7%)	2 (3.3%)	3 (3.6%)	0.95
Technical	0 (0%)	0 (0%)	3 (3.3%)	0.07
Length of ICU stay (days)				
Survivors	11 (8–15)	5 (5–5)	16 (11–29)	**0.007**
Deceased	6 (2–9.5)	1 (1–1)	3 (2–8)	** < 0.001**

Highlighted in bold are the values which are statistically significant (less than 0.05)

*ECLS therapy indicated during hospitalization for arrhythmic storm with cardiogenic shock

**ECLS therapy indicated for refractory OHCA (ECPR)

***Bleeding complications were assessed based on Thrombolysis in Myocardial Infarction classification under “major” category, defined as any intracranial hemorrhage (excluding microhemorrhages < 10 mm), fatal bleeding directly resulting in death within 7 days or overt bleeding associated with a decrease in hemoglobin concentration of 5 g/dL or a 15% absolute decrease in hematocrit

*ACLS* advanced cardiac life support, *ECLS* extracorporeal life support, *ECPR* extracorporeal cardiopulmonary resuscitation, *ICU* intensive care unit, *MODS* multiple organ dysfunction syndrome, *NA* not applicable, *PCI* percutaneous coronary intervention, *ROSC* return of spontaneous circulation, *TTM* target therapeutic management, *UNK* unknown, *WLST* withdrawal of life-sustaining therapy

### Survival at 180 days

**Fig. 2:**
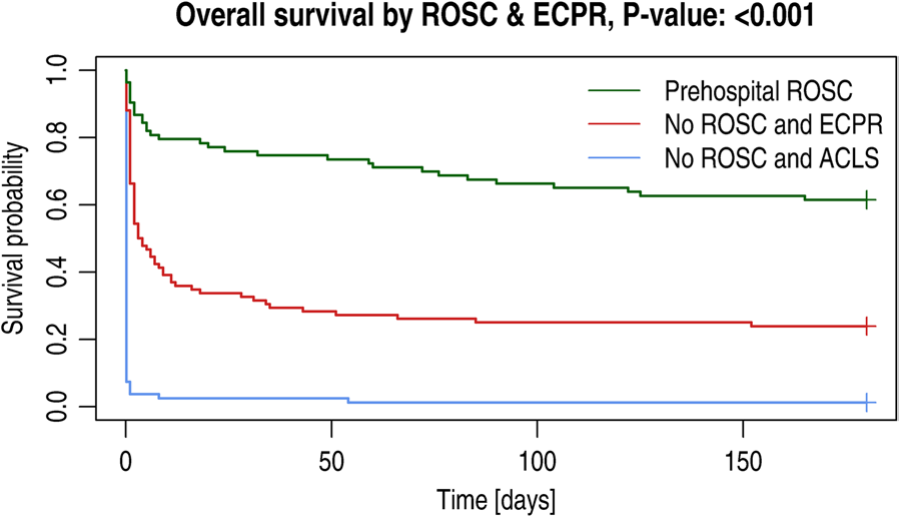
Kaplan–Meier survival curve in the study according to ROSC and ECPR status

The overall 180-day survival was 1/81 (1.2%) in patients without prehospital ROSC treated with ACLS only compared to 22/92 (23.9%) in patients without prehospital ROSC treated with ECPR and 51/83 (61.5%) in patients with prehospital ROSC (log-rank *p* < 0.001) (Fig. 2).

### Cox proportional hazards model of 180-day survival

After adjusting for the most important covariates in the Cox proportional hazards model for all 256 enrolled patients (Table 3), ECPR was associated with a lower risk of 180-day death (HR 0.21, CI 0.14–0.31, *p* < 0.001). Further, prehospital ROSC status was the strongest factor of 180-day survival in the study (HR 0.10, CI 0.06–0.16, *p* < 0.001). In addition, shockable initial rhythm, younger age and shorter time of resuscitation were all significantly associated with better 180-day survival (Table 3).

**Table 3:** The Cox proportional hazards model for 180-day mortality

Factor	Hazard ratio	95% CI	*P* value
Sex (female)	0.89	0.6–1.3	0.55
Age (per year)	1.02	1.01–1.03	**0.008**
Initial rhythm (PEA/Asystole)	2.19	1.59–3.0	**< 0.001**
Prehospital ROSC (yes)	0.10	0.06–0.16	** < 0.001**
Collapse to EMS arrival (per minute)	1.02	0.99–1.05	0.22
CPR time (per minute)	1.01	1.01–1.02	** < 0.001**
Place of cardiac arrest (public)	1.01	0.72–1.42	0.95
Successful PCI (yes)	0.77	0.52–1.12	0.18
ECPR (yes)	0.21	0.14–0.31	** < 0.001**

Highlighted in bold are the values which are statistically significant (less than 0.05)

*CPR* cardiopulmonary resuscitation, *CI* confidence interval, *ECPR* extracorporeal cardiopulmonary resuscitation, *EMS* emergency medical service, *ROSC* return of spontaneous circulation, *PCI* percutaneous coronary intervention, *PEA* pulseless electrical activity

### Neurological outcome at 180 days

Favorable neurological outcome of CPC 1 or 2 at 180 days was achieved in 1/81 (1.2%) in patients without prehospital ROSC treated with ACLS only, 20/92 (21.7%) in patients treated with ECPR and 47/83 (56.6%) in patients with prehospital ROSC (*p* < 0.001) (Table 4). Patients with an initial shockable rhythm had a better neurological outcome compared to patients with non-shockable rhythms in the prehospital ROSC (69.8% vs 15%) and ECPR group (33.3% vs 2.9%) (Table 4). Only 2/22 (9.1%) survivors in the ECPR group and 4/51 survivors (7.8%) in the prehospital ROSC group had poor neurological outcome (CPC 3 or 4) at 180 days (Table 4).

**Table 4:** Neurological outcome at 180 days according to the groups and initial rhythms

Parameter	Prehospital ROSC (n = 83)	No ROSC and ACLS (n = 81)	No ROSC and ECPR (n = 92)	*P* value
Good neurological Outcome CPC 1 + 2	47 (56.6%)	1 (1.2%)	20 (21.7%)	** < 0.001**
Initial VF	44/63 (69.8%)	0/36 (0%)	19/57 (33.3%)	** < 0.001**
Initial PEA/Asystole	3/20 (15%)	1/45 (2%)	1/35 (2.9%)	0.07
CPC of 180-day survivors				
CPC 1	44 (86.3%)	1 (100%)	18 (81.8%)	0.91
CPC 2	3 (5.9%)	0	2 (9.1%)	
CPC 3	2 (3.9%)	0	0	
CPC 4	2 (3.9%)	0	2 (9.1%)	

Highlighted in bold are the values which are statistically significant (less than 0.05)

*ACLS* advanced cardiac life support, *CPC* cerebral performance category, *ECPR* extracorporeal cardiopulmonary resuscitation, *PEA* pulseless electrical activity, *ROSC* return of spontaneous circulation, *VF* ventricular fibrillation

## Discussion

In this secondary analysis of the randomized refractory OHCA trial, ECPR increased both 180-day survival and favorable neurological outcome in patients without prehospital ROSC compared to patients treated with prolonged conventional ACLS only. In a multivariate Cox regression analysis, the use of ECPR was significantly associated with 180-day survival. This result is further supporting ECPR as an increasingly used method for r-OHCA and is consistent with previously published observational studies as well as the one randomized trial [7, 11–15].

Although proper selection of patients who will benefit from ECPR is essential, to date there is no consensus about the criteria for starting intra-arrest transport and implementing ECPR [5, 6]. In addition, significant differences in ECPR protocols between cardiac arrest centers exist [5, 7, 8] and currently published data regarding predictors of survival in r-OHCA were based on evidence from observational studies only [17, 18]. The results of multivariate analysis in this study indicate that prehospital ROSC, shockable initial rhythm, shorter time of resuscitation as well as younger age are all positively associated with 180-day survival in r-OHCA confirming findings from observational studies and systematic reviews [15–19]. However, further research is needed to achieve consensus regarding optimal ECPR strategy as excluding certain subgroup of patients without sufficient data may inappropriately limit patient care [20]. The same is true for ECPR timing as too early transport may decrease chances of achieving prehospital ROSC [4, 19], a major determinant of survival, but later transport and longer low flow time are associated with decreased survival despite ECLS implantation [15, 19]. The most relevant finding regarding the intra-arrest transport timing is derived from an observational study suggesting that ECPR should be considered between 8 and 24 min of professional on-scene resuscitation, with 16 min balancing the risks and benefits of early and later transport [19]. Prague OHCA was the first r-OHCA trial randomizing patients prehospitally during ongoing CPR in the field [8]. Patients were randomized on average after 25 min of ongoing OHCA including 15 min of ACLS, reflecting a truly refractory cardiac arrest [8]. Despite that, almost one-third of enrolled patients in the invasive arm still achieved sustained ROSC prehospitally, en route or immediately after admission, most of them having initial shockable rhythm. This highlights the need of continuous high-quality ACLS during transport to cardiac arrest center and also the urgency of further research in this area as the key question (whether conventional CPR non-responders and candidates for ECPR can be identified early during CPR) remains unanswered.

The overall 180-day survival rate for patients treated with ECPR was 23.9% in this study, which is comparable to prior observational studies reporting survival rates from 12 to 33% [11–15]. It is lower compared to the results of the ARREST trial where 6 out of 14 patients survived (43%) as this study included patients with an initial shockable rhythm only [7]. Nonetheless, survival rate of patients with an initial shockable rhythm in the invasive arm of the Prague OHCA study was actually 48.6% [8] corresponding to the ARREST trial. The Prague OHCA trial also provided randomized data confirming a vast difference in r-OHCA outcomes between patients with initial shockable and non-shockable rhythms [8]. Results of this secondary analysis further confirm poor outcomes of non-shockable rhythms despite ECPR treatment. These findings are supporting current clinical practice in many systems which limit ECPR service to patients with an initial shockable rhythm [7, 15]. Moreover, our results confirmed that patients without prehospital ROSC have very low chances to survive even with prolonged (median time 66 min) conventional ACLS without ECPR which is in line with previous findings [3, 4, 7, 13]. Based on the current evidence from observational study [15] as well as the randomized trials [7, 8], it is obvious that the subgroup of patients with an initial shockable rhythm and prolonged CPR over 45 min benefit most from the ECPR approach [8]. However, it is important to underline that ECPR must be considered early and provided in a well-established system with close cooperation between EMS and ECPR cardiac arrest center to achieve good outcomes [7, 8] as survival rates lower than 4% were reported in patients transported without field ROSC from observational studies [2, 3].

Further, almost all randomized patients in both prospective ECPR studies [7, 8] had witnessed arrest with high rate of bystander CPR which is another important prerequisite for good outcomes. Moreover, only 6% of all OHCA patients were enrolled in the Prague OHCA trial which is in line with previous reports [21, 22]. This confirms that ECPR is not a substitute for conventional ACLS but rather complementary method for properly selected refractory OHCA patients provided in the well-organized system [7, 8]. Continuous efforts to achieve maximum rates of bystander CPR are extremely important as these are associated with favorable long-term outcomes and may also increase the pool of patients considered for ECPR [7, 8, 23].

In addition, the current analysis confirms high rates of bleeding complications associated with invasive approach and ECPR [2]. Bleeding is an important limitation of ECLS therapy in all indications, especially among ECPR patients who underwent prolonged resuscitation attempts. Despite the substantial rates of bleeding, these complications were the leading cause of death in a small proportion of ECPR patients in our study (4.2%).

Neurological outcome results at 180 days in this study revealed that majority of survivors had good neurological outcome (mainly CPC 1), and only few patients treated with ECPR survived 180 days with a poor neurological outcome, similarly to patients treated conventionally. However, brain death was the third most common cause of death in the study, and irreversible brain damage is a major barrier to achieve better outcomes in refractory OHCA [24]. Further, data regarding long-term outcomes and quality of life in ECPR survivors are scarce [25] and more information is needed.

### Limitations

The present study has several limitations. First, this was a secondary analysis of the randomized trial and despite adjusting for covariates in multivariate analysis, there might have been other uncontrolled confounding variables influencing the results. Second, this is a single‐center study with limited enrollment. Third, these are the results of tertiary cardiac arrest center with considerable ECPR experience located in the urban area and the study included selected refractory OHCA population which limits the generalizability of our results.

## Conclusions

In this secondary analysis of the randomized r-OHCA trial, ECPR was associated with improved 180-day survival in patients without prehospital ROSC. Initial shockable rhythm, younger age and shorter time of resuscitation were all associated with better 180-day survival in r-OHCA. Majority of r-OHCA survivors treated by ECPR had good neurological outcome at 180 days.

## Data Availability

The datasets used and analyzed during the current study are available from the corresponding author on reasonable request.

## Abbreviations

ACLS: Advanced cardiac life support
CAG: Coronary angiography
CPC: Cerebral performance category
CPR: Cardiopulmonary resuscitation
ECPR: Extracorporeal cardiopulmonary resuscitation
ECLS: Extracorporeal life support
EMS: Emergency medical service
OHCA: Out-of-hospital cardiac arrest
PCI: Percutaneous coronary intervention
r-OHCA: Refractory out-of-hospital cardiac arrest
ROSC: Return of spontaneous circulation
TTM: Targeted temperature management
